# On the Contagious Disease of the Silk-Worm, and Its Analogy to Syphilis in Man

**Published:** 1874-07-15

**Authors:** 


### MEDICAL EXAMINER.

A

*Semi-Monthly Journal of Medical Sciences.*

EDITED BY N. S. DAVIS, M.D., AND F. H. DAVIS, M.D.

No. XIV. CHICAGO, JULY 15, 1874. VOL. XV.

### Original Communications.

### ON THE CONTAGIOUS DISEASE OF THE SILK-WORM, AND ITS ANALOGY TO SYPHILIS IN MAN.

IN the year 1865, Pasteur was instructed by the French Minister of Agriculture, to specially investigate and report upon the diseases incident to silk-worms. During the interval between the years 1853 and 1865, these disorders had reduced the annual production of cocoons in France, from sixty-five to ten millions of pounds. In the admirable work which resulted from his laborious researches,* the author remarks: "Certain disorders of the human race are accompanied by spots upon the skin, which originate in consequence of various alterations of the intestinal canal. This is not the sole observation applicable to human pathology which the experiments detailed in this work will suggest to the intelligent reader."

Diseases of the higher and lower orders of the animal kingdom are undoubtedly subject to similar conditions, in their genesis, resolution or fatal issue. It is more logical, as well as more consonant with scientific method, to observe the uniformity of a pathological law in the caries of an elephant's dentine, and the gangrene of a spider's foot, than to seek with Huxley for a community of protoplasm between the finner whale and the fungus upon a fly. The ciliae of the vorticella and of the human bronchi are not identical in structure, but they move in obedience to a similar impulse.

* Etude sur la Maladie des Vers A Soie, moyen pratique assure de la combattre et d'en prevenir le retour, par M. L. Pasteur, Membre de l'Institut Imperial de France, et de la Societe Royale de Londres. Paris: 1870. This work is the source from which have been obtained all the facts relative to the contagious disease of the silk-worm, to which reference is made in this paper.

A medical friend once remarked to the writer of these pages, that the periodical visitations and ravages of insects, presented a striking analogy to the recurrence and devastation of epidemic diseases. It is well worth the investigation to enquire if they be not alike dependent upon similar hygrometric and thermometric conditions of the soil and atmosphere.

It is proposed here to point out the analogies which exist between the pebrine of the silk-worm and syphilis in man, not because these analogies might be so interpreted as to indicate that the two disorders have, in common, a parasitic origin. It is because the knowledge we at present possess relative to contagion, is so scanty, that it may be said every new observation of its phenomena, stimulates the belief that that which is unknown and yet knowable, is largely in excess of that which is known regarding it.

Bumstead, referring to this subject in a recent paper,* says:

"The fact is, that a new field for investigation and experiment has been opened, which no one has as yet fully explored, and no one can pretend to understand. The exploration of this field promises to throw light, not only upon syphilis, but upon other contagious diseases, and even to add to our knowledge of the nature of specific poisons in general; but the work is yet undone, and any conclusions at this time are only premature."

It is preferable to select syphilis for the study of the analogies referred to above, first, because it is a disease produced by a tangible virus; and, second, because of the multiformity of its results. It is possible to secure upon the point of the lancet a drop of matter, which we can prove to be capable of producing all the complications of the disease. This is also true of pebrine. While we have, however, an equal opportunity of isolating the *materies morbi* in vaccinia, variola, malignant pustule, and certain other maladies, the polymorphism of the results produced, is not equally marked as a basis for comparison.

It is, perhaps, proper to admit, at the outset, that the investigations of Professors Stricker and Kobner, have completely exploded the theories of Lostorfer, Salisbury, and others, as to the existence and causality of *crypta syphilitica*. We have no additional information which would warrant us in reviving such dead issues. That is not the purpose of this paper. It is here intended merely to exhibit a general agreement between the origin and evolution of two contagious diseases, existing in two widely-separated orders of animals, in order that the classical feature of contagion in an extended area may be better appreciated.

The silk-worm, as is well known, is the larve of the Bombyx Mori, which deposits an ovum, from which, in turn, the caterpillar is produced. The latter, after undergoing four (in some races, three) distinct changes of integument, becomes a pupa, or chrysalis, and surrounds itself with the silk cocoon. From this, lastly, the perfect moth—imago of naturalists—effects its escape. When it is considered that, in a period of between thirty and thirty-five days, the caterpillar increases in size till it becomes eight or ten thousand times larger than the

* Am. Jour. of the Medical Sciences, April, 1873.

newly-escaped larve, it will be seen that organic life is displayed with unequalled activity in its development. Diseases, therefore, cannot but progress, *pari passu*, with an intensity proportioned to the energy of the vital forces.

In the human economy, of what paramount importance to its conservation, are the critical phases of the first and second dentition, the arrival of puberty, and the change of the menopause! In the silk worm, no less than seven equally important crises occur, during a comparatively short interval—the cycle of a brief existence, whose momentous stages offer unusual facilities for the encroachment of disease.

It is to be remarked, if we begin with the earliest phases of the two disorders, that:

1. *Pebrine and Syphilis are alike producible by artificial inoculation.* Pasteur produced a liquid capable of inoculation, by bruising a diseased worm and mixing the mass with a small quantity of water. A number of worms were selected, carefully examined in order to ensure their soundness, and thoroughly cleansed by washing, so that no germs might remain in contact with the skin. He then made a small puncture in one of the posterior rings of the body of each, and inoculated the wound by inserting into it a needle dipped in the infecting liquid. The wounds readily cicatrized, and nothing but a black or dark colored spot was soon visible in the site of the puncture. Of twenty worms inoculated in this manner, on one occasion, seven became diseased to such an extent as to exhibit from fifty to two hundred of the corpuscles characteristic of pebrine, in one microscopic field. The experimenter explains why no larger proportion of successful inoculations was made: "The blood which escapes from the wound does not invariably permit of penetration by the corpuscles which are intended to produce infection." Audoin is said to have observed the same fact in his inoculations. Many an inexperienced physician has failed of successful vaccination for a similar reason.

It should be stated, however, that most frequently pebrine is produced by the ingestion of corpuscular germs, when the worm is feeding upon the mulberry leaf. The corpuscles are then found distributed over the surface of the leaf in debris; and a single repast is said to be sufficient to occasion the disease. It is worthy of note that an intestinal lesion is then produced.

It cannot be doubted that chancres would in like manner result, if, by any natural process, the secretion from similar sores could be applied to the mucous surface of the intestines. But it may well be doubted if this species of infection of the primæ viæ, ever occurs in the human subject. A vacciniculturist of this city, however, once informed the writer that he was in receipt of numerous orders from practitioners of the homœopathic delusion, who desired to secure an infinitessimal quantity of vaccine virus, rubbed up with sugar of milk for internal administration!

2. *Pebrine and syphilis are alike communicable by accidental inoculation.* Pasteur discovered numerous cicatrices in healthy worms, which resulted from wounds. These wounds were inflicted by hooklets attached to the anterior organs of locomotion, in those caterpillars with which they had come into frequent contact. These were never seen in isolated individuals. He remarks that, not infrequently, these sharp hooklets, by which the caterpillar is enabled to cling to the leaf upon which it feeds, are inserted into the fæces or integument of diseased worms, and subsequently into the bodies of those that are sound, thus serving to propagate the disease by accidental inoculation. It is evident that there is here, also, the possibility of the production of *mediate* contamination, the porte-virus (if it be allowable to coin a suggestive word,) being exempt from infection.

3. *Pebrine and syphilis alike require a period of incubation, before the phenomena of general disease appear.* Pasteur discovered that after accidental or artificial inoculation, and also after the ingestion of disease germs, a period of from ten to twelve days elapsed before external manifestations of pebrine appeared. By feeding a number of larves, with the solution which has been already referred to, and by killing and carefully examining a fixed number of bodies at consecutive dates, he was enabled to follow the evolution of the disease, and to trace its natural history. In every instance the period of incubation was noted. This is such a constant concomitant of contagious diseases, that it may well be considered essential to their full development.*

4. *The first general indications of constitutional disease in pebrine and syphilis appear as integumentary lesions.* In the course of the experiments conducted by Pasteur, whenever a number of larves were selected for inoculation or infection, a similar number, of the same age and habitat were set aside in a healthy condition, in order to serve the purposes of comparison. At the expiration of the period of incubation referred to above, a very sensible inequality was noticeable in these two classes. Those which were left uninfected, displayed unmistakable evidence of greater well-being; while the diseased worms, when examined by the aid of a lens, exhibited numerous excessively small spots or maculæ, hitherto unnoticeable, about the head and rings. These lesions did not at first indicate the presence of the characteristic corpuscles in the skin. The "extension of the latter from centre to circumference had not yet affected the external organs. These surface-spots," says Pasteur, "only occur when the internal skin, if I may be allowed the expression, is affected to such a degree as to seriously interfere with the functions of digestion and assimilation."

Subsequently, however, integumentary lesions were produced which, upon careful examination, were found to contain the pebrine corpuscles. It is difficult to recognize the distinction here established, and not recall the difference between those superficial syphilides, which disappear readily under appropriate treatment, and those which contain a specific morbid product. One instinctively recurs to the theory of Jonathan Hutchinson and others, that the lesions of secondary syphilis are febrile phenomena. These precede the deposits of tertiary forms, in which the "still-born" product of Lancereaux is to be distinguished.

The patches upon the integument

* See *Nouv. Dict. de Med. et de Chi. Jaccoud, Art. Contagion.*

in pebrine are generally of a dark color, sometimes black, (whence the name,) some more and some less clearly defined. The petechial character of this stage of the disease has given it the name by which it is known among the Italians (Petechia of the Silk-Worms). When completely developed, these stains are surrounded by a yellowish areola, which exhibits various gradations in color. Sometimes they constitute the sole symptoms of the disease.

M. Quatrefages, with whose opinions Pasteur is not in complete accord, declares that the alterations, described above, are best studied in the skin of the young larves. In these he could occasionally descry nothing more than a yellowish tint, slightly obscuring the hyaline transparency of the tissues. Somewhat later, a darker stain became visible, shading gradually into brown, until the translucence of the epidermis was lost. Finally, a brownish-black stigma remained, which was accompanied by a disappearance of all traces of organization. About this, as a nucleus, a yellowish areola extended, which, in his opinion, marked the invasion of the surrounding tissues. This process generally continued until arrested, either by the death of the worm, or by the regular replacement of the old, by a new integument. In the course of two or three days, however, the new cuticle, which at first appeared entirely normal, was in its turn affected by the disease, "proving," says Quatrefages, "that the lesions were not local phenomena, but signs of a constitutional malady, dependent upon a profound cause."

Pasteur has noted that the development of the pebrine corpuscles proceeds with an unexampled rapidity during these periods of metamorphosis—a circumstance which our knowledge of the laws of pathology would lead us to expect. He disagrees with Quatrefages in the supposition that the integumentary lesions are localized foci, from which a quasi-gangrenous process extends to the invasion of adjacent tissue; but considers each stigma to be a resultant of corpuscular development, and the changes in the appearance of the maculæ, not due to molecular death, but to neoplastic hyperplasia.

In addition to the symptoms noted above, certain other indications of disease are described in the adult moth, as, for example, vesicles, varices and bullæ filled with a sanguinolent fluid, under or near the wings. Some of these were observed to burst, and their contents, escaping and drying, were found to form adherent crusts, black and viscous, of the size of a pea.

5. *Pebrine and Syphilis are alike productive of a specific adenopathy.* The secretion of the silk glands of the pupa has solely contributed to the value placed upon the insect by the commercial world. In a pathological point of view, these glands possess especial importance from the fact that they are rapidly affected in pebrine. The large pentagonal cells which surround the canal where the silk is secreted in a viscous state, exhibit in a diseased condition numbers of oval corpuscles, crowded together, and sometimes collected in such masses that they lend an appearance of hypertrophy to the glandular tissue. Viewed with a low power, they exhibit whitish projections brilliant in color, of oval form, and very clear definition. They are, without doubt, evidence of the extension of the disease to the visceral organs of the worms: and the total incapacity of the larves to produce cocoons—those of them, at least, which are profoundly affected—is a proof of the destructive agency exerted by the glandular neoplasms.

In syphilis, not only are those glands affected which are in the chain of the great system of lymphatics, but those which are actively concerned in hæmatopœsis. There is strong reason to believe that, aside from the development of hepatic gummata, usually found in the tertiary stage, one of the earliest symptoms of constitutional syphilis is dependent upon some disturbance of the glycogenic function of the liver. Dr. Charles Murchison has recently concluded,* after reviewing the discoveries of Hoppe Seyler, Bernard, Lehmann, McDonnell, Hirt, of Zittau, Weber and Kolliker, that "the glycogen secreted in the liver cell combines with nitrogen and forms an azotised protoplasm which maintains the nutrition of the blood and tissues." In this light the chloroanæmia of early syphilis is most readily explained—a condition which is constant in all but benign cases, and which constitutes an important indication for successful treatment.

6. *Pebrine and Syphilis are, alike, diseases of the blood.* In a healthy state the blood of the larve is a transparent albuminous fluid—colorless in the case of those races which produce white silk; and golden yellow in those which produce yellow silk. Under the microscope, innumerable spherical bodies appear, of various sizes, the largest of which does not in its greatest diameter exceed .0039 of an inch. They seem endowed with individual vitality, and continually reproduce themselves during the life of the insect. When the latter is infected with pebrine, the number of the blood globules decreases—thus inducing a species of chloroanæmia—and the albuminous fluid becomes charged with an immense number of minute animated corpuscles .01 of an inch in diameter, increasing in proportion to the disappearance of its normal ingredients. These are the pebrine corpuscles already described, which Pasteur is disposed to regard as the parasitic germs of a species of psorosperm. They are oval or reniform in contour, destitute of ciliæ, and move rapidly, apparently at will, sometimes advancing and sometimes receding in the vascular channel.

The genus "psorosperm" was first established by Jean Muller, after his observation of certain anomalous organisms in different varieties of fishes, and especially in the fresh-water pike. But certain later expressions of Pasteur seem to imply that his mind is not perfectly clear as to the parasitic character of the germs described by him. In some of his communications to the Academy of Sciences, for example, he uses language from which it might be inferred that the disease originated in generations of the ancestors of these worms, whose connective tissue had undergone a peculiar cell-metamorphosis.

It is well-known that Beale* adduces very strong grounds for the belief that contagious disease germs are not parasites, and his opinions are

* Lancet, June, 1874.

* Disease Germs; their nature and origin. Lionel S. Beale. London: 1872.

largely the result of researches upon the subject of the cattle-plague. Let it be supposed, in accordance with his views, that the corpuscles described by Pasteur are bioplasts—contagious living disease germs—that they are the descendants of blood or tissue bioplasts; that subsequently, either by hyper-nutrition or retrogression, they have undergone a conversion of energy, and become powerful to self-multiply indefinitely, and powerless to build up new and normal structures. This would explain the amœbiform movement of the pebrine corpuscles, their contagiousness, their virulence and their destructiveness. Not only so, but it would do away with the need of resorting to a novel species of parasite, in order to explain the phenomena. It should be stated in this connection, that Beale considers the observations of both Pouchet and Pasteur open to objections upon the ground of their employment of very low powers. Many of the germs figured by Beale were viewed with an objective of one-fiftieth of an inch focal distance, enlarging the dimensions of these organisms two thousand eight hundred diameters.

In such a field as this, speculation is illusory, and scientific deductions are alone to be desired. Still the general trend of the exposed strata is in one direction. They to whom the conservation and transmutation of forces is an unalterable fact of physics, have no difficulty in believing that there is a similar law to which the vital forces are subject. Heat, light and electricity are shown to be modes of motion—interchangeable and intercurrent. The day is, perhaps, not far distant, when it will be clear that contagious and other diseases, which betray themselves by structural lesions, depend upon the mode of motion of the bioplast. This motion is known to be the measure of its energy. Can we not even declare that it is the essential condition of its vitality? Motionless bioplasm is dead. The transmutation of a normal to an abnormal energy should, therefore, produce disease and ultimate death. If this can be shown, it will be apparent that by an inversion of this process, restoration from disease occurs.

Guerin-Meneville, in a report to the French Agricultural Society in 1849—mark the date!—gives expression to the same general thought. "It seems clear to me," said he, "that these granules (pebrine corpuscles) are the elements of new blood globules, normally produced and launched into the vascular currents of healthy worms; but in pathological conditions they lack certain essential elements, and are therefore arrested in the progress of development."

Pasteur describes the mature corpuscles as brilliant of refraction and ovoid in shape. They subsequently become pyriform, surround themselves with a double envelope, and exhibit a slight flattening at the narrower extremity. They contain granules, either free or adherent to the cell wall, and these, he believes, after their exit by rupture of the cell envelope, serve as new centres for the development of new corpuscles, and thus extend the disease. The tissue of these organisms was supposed to contain sarcode.

7. *Pebrine and Syphilis are hereditary disorders.* The transmission of the disease of the silk-worm from one generation to another, has been the most fruitful source of evil in the propagation of the species. Unfortunately, before the microscope had been employed in the study of the malady, sericulturists could not be persuaded to believe that apparently healthy ova from parents of equal apparent health, contained the seeds of the devastation which had blasted their hopes of profit for the preceding year. Such, however, has been too frequently the case; and the success of Pasteur in totally eliminating the disease from those nurseries in which his method was pursued, was due to his recognition of this fact. It is not a little remarkable in this connection, to observe that:

8. *In Pebrine, as in Syphilis, when one parent only is affected with the disease, healthy offspring may be produced.* This general fact was demonstrated by a great number of experiments upon the coupling of moths, in which there was undoubted evidence of corpuscular disease either of the male or the female. It appeared, also, from these experiments, that ova entirely sound were generated occasionally by males who exhibited very extensive traces of the malady, when assorted with females who, while they were indubitably infected, yet exhibited very few of the pathognomonic lesions of pebrine. The experimenter explained these circumstances by the conditions incidental to the chrysalis. If the latter became infected with pebrine so as to exhibit corpuscles very soon after the formation of the cocoon, the moth and its ova were almost certain to be similarly diseased. But if this development did not occur until near the time for the escape of the imago, then the ova of the moth might be entirely sound. In the case of the syphilitic ovum, similar results are said to be declared, according as infection occurs early or late in utero-gestation.

Other analogies between these diseases obviously exist which might be in turn the subject of comment. Such, for example, are the involvement of the nervous system and centres in each—the infecundity of infected females who are liable to sterility and the production of blighted germs; the non-inoculability of the infectious matter obtained during the later stages of each disease, and the liability of each to complication by the advent of other maladies.

It should be stated that Pasteur himself is disposed to regard pebrine as analogous to pulmonary phthisis. But he is careful to announce that in establishing a resemblance between the facts which he has observed and those relative to diseases of the human race, he does not speak as an expert.* The hereditary influence of phthisis seems to have attracted his attention to this subject.

But there are many objections to this view founded upon the clinical history of terberculosis. This latter disease is neither infectious, contagious nor inoculable.† Nor does it

* "Je desire toutefois que l'on sache bien que je parle en profane, lorsque j'etablis des assimilations entre les faits que j'ai observes et les maladies humaines." T. 2 p.

† Bouillaud states that, "the tuberculous virus is an hypothesis which up to the present time rests upon no exact nor trustworthy observation; and there does not exist a single instance of tuberculosis of the lungs, or of any part of the body, being produced in the human species by means of specific (viruproduce a pathognomonic cutaneous lesion.

It is true, as stated by Pasteur, that children born of phthisical parents, may, in some instances, merely become more or less sickly, while in others tubercle may be developed in different degrees at various ages. But one has not to consult the statistics of consumption in order to establish this diversity in the evolution of hereditary disease. Congenital syphilis may infect the ovum, the fœtus at term, and the infant newly born or which has survived for weeks and months. But this is not the limit of its effect. Massa narrates cases in which the disease was developed between three and eleven years of age; Balling, similar instances at the age of sixteen; Rosen, at eleven; Baumes, at four; Cazenave, at eighteen; Fournier, at twenty-five; Zambaco, at twenty-six.* Other authors cite cases which illustrate the same point. In the face of these observations who will venture to say: "Thus far doth it come, and no farther?"

In concluding the consideration of the general subject here discussed, few will refuse to concur with the opinions expressed by Dr. William Aitken. "The diseases of the lower animals," says this author, "rarely form any part of the study of the student of medicine. The diseases of plants are almost entirely neglected. Yet it is clear that until all these have been studied, and some steps taken to generalize the results, every conclusion in pathology regarding the nature of diseases must be the result of a limited experience from a limited field of observation."

J. N. H.

lent) inoculation." As to contagion, the experiments of Erdt, Villemin, Simon, Herard and Clarke have been shown by Lebert, Nyss, Sanderson and Fox, to demonstrate merely the irritative character of subcutaneous injections of putrid matter.

* Lancereaux; Traite de la Syphilis. Paris: 1866.

---

DRUNKENNESS AND INSANITY.—The last English census report says that it has been established by the observation of many authorities that intemperance is the most prolific cause of insanity, especially among the working classes. To the cases of madness resulting from habits of drunkenness on the part of the individuals themselves must be added the numerous instances in which persons owe their insanity to the intemperate habits of their parents. It is said that the fruitful source of mental disease, hereditary taint, insanity inherited from parents, is fostered by the insane being allowed to propagate their kind with scarce any effort to check so deplorable an event. Large numbers of the insane and the idiotic still remain at home, or are "boarded out," and become in many instances the agents of extending the fell malady through their offspring.—*Philadelphia Med. and Surg. Reporter.*

